# From cognitivism to autopoiesis: towards a computational framework for the embodied mind

**DOI:** 10.1007/s11229-016-1288-5

**Published:** 2016-12-22

**Authors:** Micah Allen, Karl J. Friston

**Affiliations:** 1 Institute of Cognitive Neuroscience, University College London, London, UK; 2 Wellcome Trust Centre for Neuroimaging, Institute of Neurology, University College London, 12 Queen Square, London, WC1N 3BG UK

**Keywords:** Predictive processing, Embodied cognition, Interoception, Active inference, Connectionism, Computationalism, Enactivism

## Abstract

Predictive processing (PP) approaches to the mind are increasingly popular in the cognitive sciences. This surge of interest is accompanied by a proliferation of philosophical arguments, which seek to either extend or oppose various aspects of the emerging framework. In particular, the question of how to position predictive processing with respect to enactive and embodied cognition has become a topic of intense debate. While these arguments are certainly of valuable scientific and philosophical merit, they risk underestimating the variety of approaches gathered under the predictive label. Here, we first present a basic review of neuroscientific, cognitive, and philosophical approaches to PP, to illustrate how these range from solidly cognitivist applications—with a firm commitment to modular, internalistic mental representation—to more moderate views emphasizing the importance of ‘body-representations’, and finally to those which fit comfortably with radically enactive, embodied, and dynamic theories of mind. Any nascent predictive processing theory (e.g., of attention or consciousness) must take into account this continuum of views, and associated theoretical commitments. As a final point, we illustrate how the Free Energy Principle (FEP) attempts to dissolve tension between internalist and externalist accounts of cognition, by providing a formal synthetic account of how internal ‘representations’ arise from autopoietic self-organization. The FEP thus furnishes empirically productive process theories (e.g., predictive processing) by which to guide discovery through the formal modelling of the embodied mind.

## Introduction

Recent developments in cognitive science and neuroscience have led to a growth of interest in “predictive processing” theories of mind and cognitive function (Clark 2013; Friston 2010; Hohwy 2013). These diverse approaches share a common ground in situating top-down, error-minimising predictions as the key locus of information processing, as opposed to more classical accounts which emphasize feed-forward feature recognition (Marr 1982). Of these, some predictive approaches are strictly cognitivist, emphasizing internal, modular representations. Other more recent variants endorse connectionism to varying degrees. Perhaps counter-intuitively, predictive processing has also appealed to concepts and mechanisms from embodied, enactive, and dynamical systems theory approaches; particularly those that come under the rubric of [en]active inference. While this plurality of approaches is a desirable product and facilitator of scientific discourse, it can also lead to theoretical ambiguity. For example, attempts to derive ‘predictive processing’ theories of consciousness, attention, or social cognition are likely to diverge strongly depending on which variant and associated commitments are taken as given; especially if there is erroneously assumed to be one singular account of prediction in the brain. Here, we attempt to provide a pacific overview of how these myriad approaches can be differentiated in terms of their commitment (or lack thereof) to the embodied and enactive mind. Furthermore, we argue that Active Inference, as entailed by the Free Energy Principle (FEP), is not only fundamentally enactive and embodied, but further offers a synthetic, empirically productive resolution to long-standing disagreements between internalist and externalist viewpoints.

## From modularity to the dynamic mind

The ability to predict future (sensory and embodied) states is essential for the efficient control of perception and action. This notion has a long history in cognitive science; for example, Helmholtz first suggested that to localize visual stimuli, the brain calls upon an efference copy of oculomotor commands to predict gaze-position, rather than by relying on the sensation of the ocular orbit^1^ (Miall and Wolpert 1996; Wolpert and Flanagan 2001). Helmholtz then demonstrated this principle by performing a simple experiment on his own sensorium, in which he found that pressing on his eyelid could generate a false sensation of motion. Helmholtz reason that this simple trick worked because it moved the eye without engaging the ocular muscles, causing the efference copy of eye position to incorrectly signal that the world had moved . Thus, our perception of the world was as much inference as sensation.^2^

**Fig. 1:**
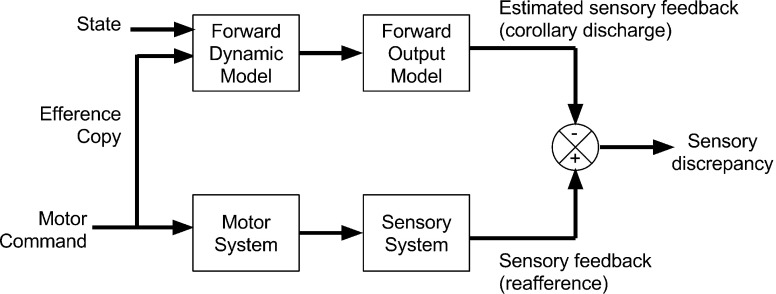
An early comparator-based predictive coding model of motor control, in which a forward model of expected sensory consequences is compared with the intended consequences of an action. The result discrepancy (‘Motor Error’) is then used to refine motor commands. Adapted from Miall and Wolpert (1996)

This notion of comparing predicted with actual sensory states to refine and control behavior has today grown into wide-reaching family of simple yet powerful ‘comparator’ models explaining motor control and awareness (Frith 2012; Miall and Wolpert 1996, see Fig. 1 below). Comparator models suggest that action initiation generates a copy of the efferent motor signal, which is passed to a specialized brain module (a forward model) to compute ‘corollary discharge’; i.e., a predicted sensory state if the motor command were enacted. This signal is then passed to another module (a comparator), which compares the predicted outcome with actual sensory (e.g., proprioceptive and tactile) inputs, which are sometimes computed by a separate inverse-module. This simple computation can, for example, disambiguate internally vs. externally generated signals, and acts as a training signal to refine motor commands (Miall and Wolpert 1996; Wolpert and Flanagan 2001). Such models have a long history in mechanical engineering, optimal control theory, and robotics, as they elegantly and efficiently provide stable motor control under a variety of conditions (Craik 1948, 1947).

The ability of motor-comparison accounts to distinguish between externally and self-generated actions led to their widespread application in the cognitive sciences. For example, one influential proposal holds that our conscious sense of agency depends on the comparison of expected and actual states. Along these lines, Chris Frith and others (Frith 2012, 1987; Frith and Done 1989; Synofzik et al. 2008) argued that the sense of agency depends upon the interaction of two feed-forward comparators, one comparing desired and predicted states to generate a feeling of control, and another comparing predicted and estimate states to generate self-ascription. This model was further expanded to explain schizophrenic delusions of thought insertion, presumed to be driven by a disruption of the self-monitoring mechanism, i.e., the intentional and motor-comparison modules which give rise to the sense of agency.^3^

Philosophically speaking, comparator-based models are unambiguously cognitivist: information processing proceeds within the confines of the brain by compartmentalized modules, which compute sanitized representations of expected sensory and motor states. This emphasis on functional localization and modularity led directly to attempts to identify various comparators in the brain; for example, by using functional magnetic resonance imaging (fMRI) to test the hypothesis that the cerebellum or premotor cortex acts as a central comparator for motor activity (Blakemore et al. 2001, 2002; Blakemore and Sirigu 2003). Crucially, comparator models are agnostic regarding the specific mathematical computation underlying their function, which can be explained by a variety of Bayesian (e.g., variational Bayes, Kalman filtering) or non-Bayesian mechanisms (e.g., reinforcement learning) (Friston 2011; Peters and Schaal 2008; Wolpert et al. 1995). Such models are thus ultimately functionalist in nature; particular behaviours are explained by appeal to a revision of modality-specific prediction errors, as computed by encapsulated, physically localized brain modules, without specifying a particular computational implementation (Fodor 1983). This is an appealing explanatory feature from the perspective of experimental psychology and neuroscience, as cognitive functions can be dissembled into their dependence on individuated modules, as determined by the logic of pure insertion and related identification of maximal functional contrasts (Friston and Price 2011). For example, the contrast of expected versus unexpected social norm violations can hypothetically be used to reveal the putative social-norm prediction error region of the brain (Koster-Hale and Saxe 2013). Further, through factorial experimental design, hypothetical interactions between classes of predictions can be tested,^4^ allowing for nonlinearity and context sensitivity in basic predictive processing. Thus, although the elegance of comparator models has led to experimental and clinical applications across a variety of cognitive (Frith 2012), social (Kilner et al. 2007; Koster-Hale and Saxe 2013) and affective domains (Seth 2013; Seth et al. 2012), it should be clear that they are predictive-processing revision of cognitivism, rather than a radical new paradigm.

## Radical predictive processing: a connectionist approach

The predictive coding implicit in comparator models of motor (or social) control is clearly cognitivist; rich internal models, which explain a world hidden from the agent, do the functional work of cognition. Such applications of predictive coding at best ignore embodied and enactive cognition and are at worst irreconcilable.^5^ More generally, while comparator-style models provide much needed ‘guidelines for discovery’ (Chemero 2011), they do not evoke the revolutionary views espoused by philosophers like Andy Clark, who writes:

Predictive processing plausibly represents the last and most radical step in this retreat from the passive, input-dominated view of the flow of neural processing (Clark 2015, p. 2).

Indeed, the close association of comparator schemes with strictly modular internal predictions may lead many to intuit that all predictive processing approaches are inherently disembodied. Some have already argued exactly this point (Hohwy 2016). To better appreciate the revolution envisioned by Clark, one must envision a ‘massively predictive’ brain, in which neurons ubiquitously encode either predictions or prediction errors in a globally inter-connected hierarchy. Such a framework has been described by Clark and others as ‘radical predictive processing’ (Clark 2013, 2015); note that this is distinct from, though not exclusionary of the ‘Bayesian Brain Hypothesis’, which denotes the more specific ascription that not only is the brain massively predictive in nature, but also that prediction error minimization operates according to probabilistic Bayesian inference (Friston and Kiebel 2009; Knill and Pouget 2004). In either case RPP posits that a localized comparator region of the brain does not (necessarily) carry out the comparison of expected and actual sensory information for a given domain. Instead, cognition is accomplished by a canonical, ubiquitous microcircuit motif replicated across all sensory and cognitive domains in which specific classes of neurons reciprocally pass predictions and prediction errors across the global neuronal hierarchy (Bastos et al. 2012; Douglas et al. 1989). Depending on whether one subscribes to the Bayesian Brain theory, the integration of these signals may also follow the law of Bayesian inference, in which both predictions and prediction errors are weighted by their precision or confidence.^6^

Thus the move to RPP championed by Clark, Hohwy, and others revises the classical view of information processing as the passive recollection of environmental features, to instead emphasize the global top-down cascade of predictions across the neuronal hierarchy.^7^ RPP constitutes a strong form of connectionism, in which it is the overall dynamics of the nervous system^8^ that accomplish information processing rather than compartmentalized modules. Further, for Clark the predictive brain constitutes an interlocking tapestry of ‘action-oriented’ representations, which are well-poised to efficiently exploit the morphological structure of the body and immediate environment, ultimately providing a mechanistic account of sensorimotor views of perception (O’Regan and Noë 2001) and the extended mind hypothesis:

... the selection of task-specific inner neural coalitions within an interaction-dominated PP economy is entirely on a par with the selection of task-specific neural–bodily–worldly ensembles. The recruitment and use of extended (brain–body–world) problem-solving ensembles now turns out to obey many of the same basic rules, and reflects many of the same basic normative principles (balancing efficacy and efficiency, and reflecting complex precision estimations) as does the recruitment of temporary inner coalitions bound by effective connectivity. In each case, what is selected is a temporary problem-solving ensemble (a “temporary task-specific device”—see Anderson et al. 2012) recruited as a function of context-varying estimations of uncertainty. (Clark, EP p. 16)

**Fig. 2:**
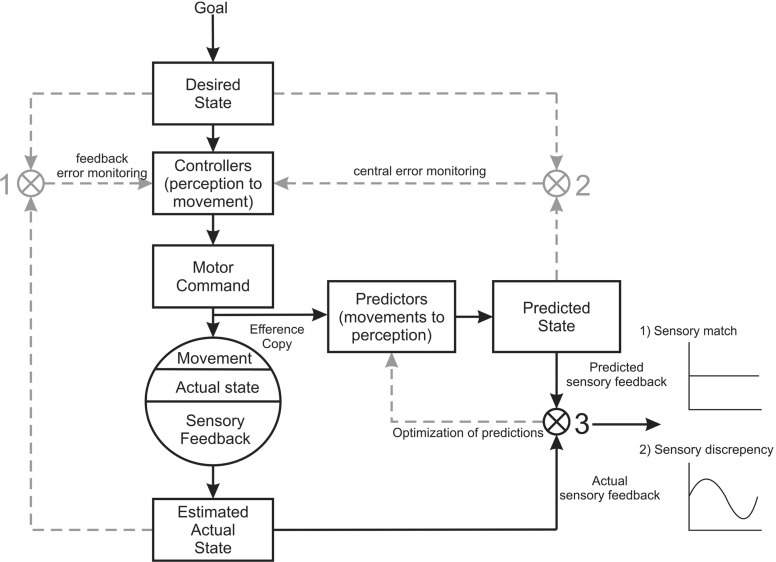
The comparator model of sense of agency, in which discrete modules compute motor, intentional, and self-related prediction errors. The central monitoring of these signals is then thought to underlie the overall sense of agency for thought and action. Adapted from Synofzik et al. (2008)

**Fig. 3:**
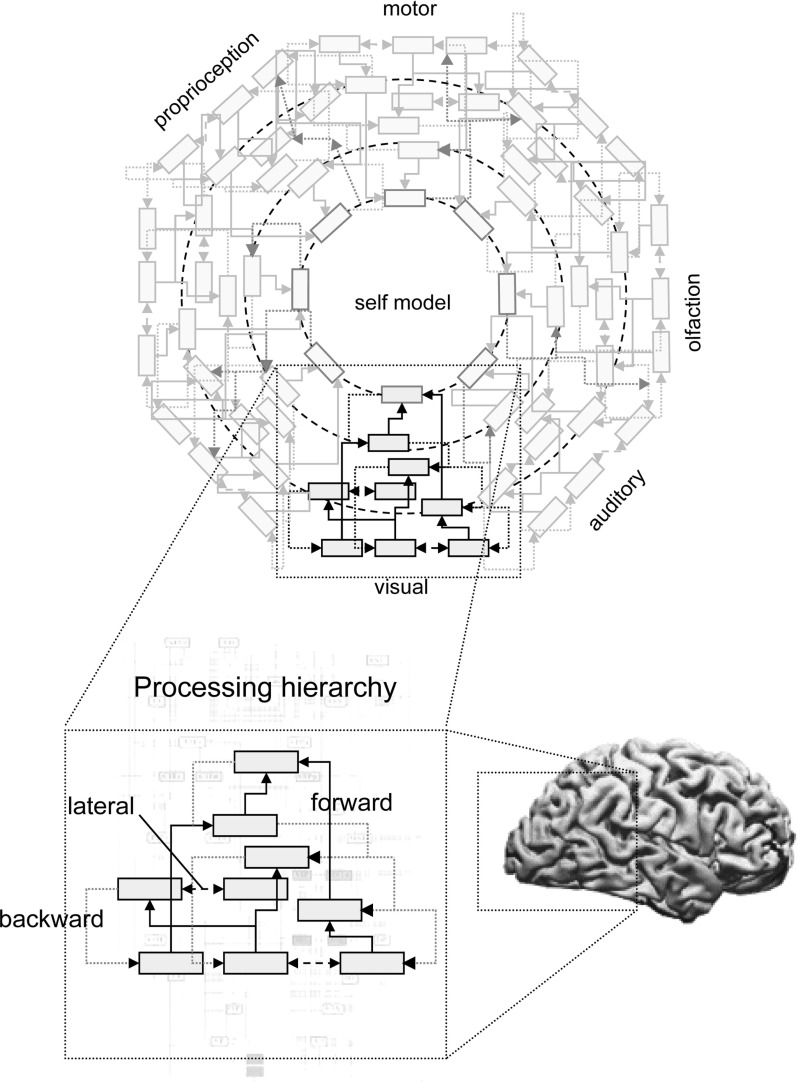
Depiction of the global, centrifugal predictive hierarchy according to radical predictive processing. Individual sub-components are distinguished by a feed-forward specialization as determined by the statistics of sensory inputs. Lateral connections and global precision-carrying signals (e.g., dopamine, norepinephrine, *highlighted in red*) link the network into a ‘centrifugal’ hierarchy with ‘inner’ and ‘outer’ layers. In some cases the ‘inner layer’ may be described as a global or self-model, which predicts the internal (visceral and neural) dynamics of the organism (adapted from Friston 2005). (Color figure online)

However, here it is worth illustrating a few points of caution. On the one hand, the fundamental explanatory locus of RPP is indeed the global minimization of prediction error. This implies that the relevant generative model is not ‘contained’ in any single neuron or module, but instead embodied in the entire pattern of connection weights as distributed across the nervous system and potentially, the body itself.^9^ This can be understood by analogy to Ashby’s ‘good regulator theorem’ which states that every good regulator (i.e., a control system which maintains integrity in the face of change) must be a model of its environment (Conant and Ashby 1970). However, even considering the above, RPP does not necessarily commit one against all functional localization or modularity. This is because individual elements of the overall neuronal network may be more or less compartmentalized according to their specific pattern and probabilistic density of feed-forward, feedback, and lateral connections; the fusiform face area may be relatively specialized for faces (or other features) in lieu of this connection asymmetry. It does however contrast in a substantive way with comparator-based formulations in the sense that the entire neuronal architecture within the brain becomes one forward or generative model with a deep hierarchical structure. Here there are no functionalist ‘goals’, ‘desired outputs’ or ‘motor commands’ (compare Fig. 3 with Figs. 1, 2); the entire system is in the game of predicting the sensorium—and nothing more (even if prediction excites physical movements through motor reflexes—see below).

To further illustrate this principle, consider the visual system, which receives specialized inputs from the retina conveying spatial patterns of visual information. Neurons at the lower levels of the visual hierarchy, in virtue of having few lateral connections to other primary sensory areas, are therefore specialized for predicting visual features. Similarly, at the highest level of the cortex, domain general, supra-modal expectations generate predictions that are unpacked hierarchically—all the way down to modality specific levels. This deep processing may provide a formal placeholder for the dissociation of personal and sub-personal mechanisms, which is sometimes levied as a critique against RPP. For example, we are notoriously poor at folk-psychological physics judgements, and our explicit judgements in this domain are typically incorrect or biased (rather than being Bayes-optimal Bowers and Davis 2012; Marcus and Davis 2013). However, even if sub-personal partitions of the hierarchy calculate (e.g.,) a Bayesian visual algorithm, higher levels may necessarily incorporate self-referential information in the form of prior beliefs or long-term memory,^10^ to produce the biases that characterize explicit (posterior) beliefs. For similar reasons, while neuromodulation of post-synaptic gain via (e.g.,) dopamine, norepinephrine, and other neurotransmitters are argued to communicate the fidelity or precision of beliefs (Feldman and Friston 2010; Friston et al. 2014a, b, 2012a, b; Kanai et al. 2015; Moran et al. 2013), individually these systems can be more or less specialized for a colloquial role in virtue of the neural partitions they interact with; for example, dopamine may modulate precision of beliefs about controllable, potentially rewarding outcomes, whereas norepinephrine may modulate sensory precision to orchestrate (endogenous) attentional selection.

Finally, it should be noted that RPP does not commit one to radical empiricism, or negate all possibility of functional nativism. As RPP emphasizes a more global, connectionist understanding of brain function, it may seem that all function must be learned in development *sui generis.* On the contrary, RPP emphasizes that it is the overall pattern of connections within and between hierarchies that constitutes the form of the generative model. This implies that genetically pre-specified connection patterns—laid down during foetal development—pre-ascribe some functional specification prior to (empirical) learning.^11^ This ultimately suggests that the nervous system is itself selected by evolution to minimize prediction error within a particular ecological niche, and may more speculatively support a role for epigenetic mechanisms in propagating cortical function from one generation to the next. Indeed, the notion that evolution is itself a predictive processing engine is receiving greater attention in theoretical biology; e.g., Fisher’s fundamental theorem and replicator formulations as Bayesian filters (Fernando et al. 2012; Harper 2011). Thus, according to RPP, prediction error unfolds not only at ontogenetic but also phylogenetic timescales; if the brain (and body) constitute a generative model, than those embodied graphs best suited to their environmental niche will be selected by evolution. In this way, nature itself minimizes prediction error by selecting organisms whose structure and morphogenesis best predicts their environment (and the actions needed to survive within it). In short, natural selection is nature’s way of performing Bayesian model selection, offering phenotypes to the environment as plausible hypotheses for explaining sensory exchange with an econiche—and selecting those that survive as the hypotheses with the most evidence (i.e., least free energy). Clearly, the enactivist stance can also be invoked at this level; especially when we consider phenotypes create their own (designer) environments, leading to a circular causality between natural (or Bayesian model) selection and the (designed) econiche that itself becomes subject to selective pressure.

## Interoceptive and embodied predictive coding

These considerations raise several important questions regarding embodiment and predictive processing. If the brain itself is taken to constitute a generative model subject to evolutionary pressure, can this metaphor be extended to the body? To what extent can predictive processing be considered ‘embodied’; can the morphology of the body, and its possibilities for action themselves be construed as an ‘embodied prior belief’ guiding inference? What roles do affective and interoceptive cues play in hierarchical inference? Can one sensibly speak of embodied cognition, and still endorse the notion that the ultimate function of the brain is to recover ‘hidden’ causes, sampled vicariously through sensory epithelia?

A bevy of recent neuroscientific and philosophical work aims to address these questions (Ainley et al. 2016; Allen et al. 2016a, b; Apps and Tsakiris 2014; Barrett and Simmons 2015; Bruineberg and Rietveld 2014; Chanes and Barrett 2016; Clark 2015; Gu et al. 2013; Limanowski and Blankenburg 2013; Seth 2013, 2014a; Seth et al. 2012). Like predictive processing itself, so-called ‘embodied’ or ‘interoceptive’ predictive coding (a.k.a. interoceptive inference) ranges in scope from straightforward extensions of comparator-based approaches (Seth et al. 2012), to treatments couched in dynamical systems theory, enactivism, and ecological psychology (Bruineberg and Rietveld 2014; Kirchhoff 2016). Although it is difficult to summarize these views under a single banner, all share a common emphasis on the importance of body-related inferences in cognition, whether to lend affective content to perception or provide a deeper understanding of the predictive mind.

An early example is found in the “interoceptive predictive coding” (IPC) hypothesis put forward by neuroscientist Anil Seth (Seth 2013; Seth et al. 2012). Seth and colleagues frame the conscious sense of presence^12^ as depending upon interactions between an interoceptive-comparator integrating ascending visceral signals^13^ with top-down autonomic control, and the motor-agency comparator previously described. Seth further argues that it is the integration of feed-forward motor and visceral expectations which generates bodily and affective experience:

... Our model proposes that presence is the result of successful suppression by top-down predictions of informative interoceptive signals evoked (directly) by autonomic control signals and (indirectly) by bodily responses. According to the model, disorders of presence follow from pathologically imprecise interoceptive predictive signals. The model integrates presence and agency... offers a novel view of emotion as “interoceptive inference”, and is relevant to emerging models of selfhood based on proprioception and multisensory integration (Seth et al. 2012, p. 2).

**Fig. 4:**
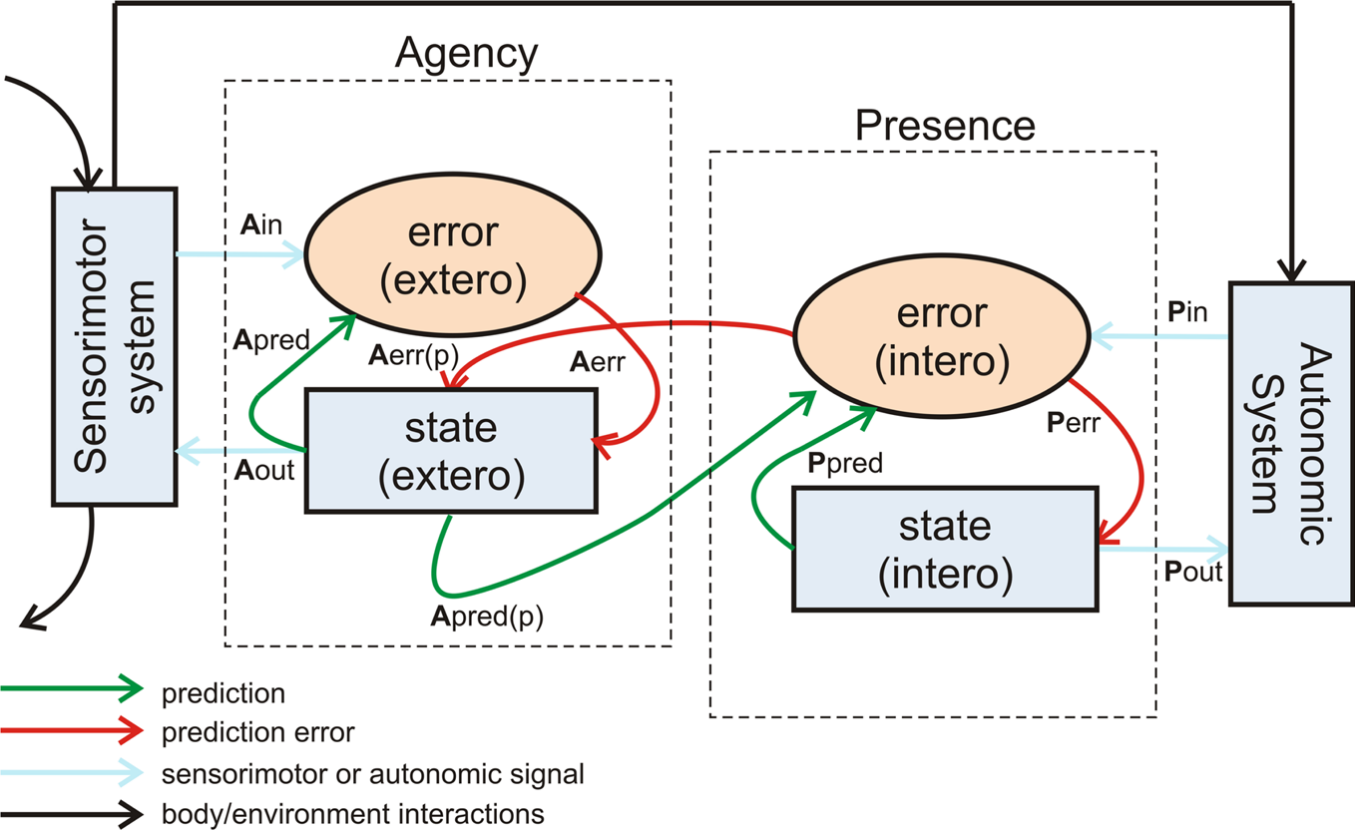
The interoceptive predictive coding model. An interoceptive and motor comparator integrating visceral and agentic signals, respectively, are combined together to produce an overall feeling of conscious agency and presence. The presence comparator is proposed to be within the insular cortex, and is also argued to underlie ‘emotional inference’. Adapted from (Seth et al. 2012)

This comparison is argued to depend upon the anterior insular cortex acting as an “[interoceptive] comparator underlying the sense of presence” (Seth et al. 2012, p. 6) in interaction with motor signals arising from the agency-comparator (see Fig. 4). This proposed role for the insula as a core module in the interoceptive hierarchy is a key motif appearing in a variety of related approaches (Barrett and Simmons 2015; Gu et al. 2013; Seth 2013). To better understand why this region is so emphasized, we briefly review the relevant neuroscience.

Anatomically, the insular cortex is a scallop-shaped structure folded deep within the lateral sulcus, where it lies nestled between the temporal, parietal, and frontal lobes. Convergent functional, connectivity, and cytoarchitectonic evidence suggests that the region is important for integrating a wide range of bottom-up sensory inputs with top-down predictions or control signals (Allen et al. 2016a; Klein et al. 2013). When the physiologist Constantin Von Economo first mapped the region’s cellular anatomy, he found a posterior zone with densely concentrated granular cells (specialized for integrating diverse inputs), and an agranular anterior zone dense with ‘Von Economo’ neurons (specialized for modulating distant cortical areas) (Gu et al. 2013; Klein et al. 2013). He further noted that no obvious demarcation between the posterior and anterior zones could be found. Instead, the two areas merged together in a continuous gradient from one cell-type to another, suggesting an integrative function of the region. This notion of the insula as integrating across the hierarchy is now further supported by functional activation and connectivity studies (Cerliani et al. 2012; Margulies et al. 2016), which also demonstrate a continuous gradient from multisensory and embodied input-integration to complex behavioural regulation as one moves along the posterior-to-anterior axis. Thus, whereas the posterior insula exhibits mostly multi-sensory responses^14^ and is broadly connected to thalamic and primary sensory regions, the anterior insula is instead responsive to attentional (salience, response inhibition) and affective (emotion regulation and awareness) conditions and sends projections to the parietal-frontal control regions and brainstem nuclei (Allen et al. 2016a; Cerliani et al. 2012; Klein et al. 2013; Uddin 2015), with both profiles being freely mixed in the middle insula. Interoceptive predictive coding thus argues that the insular cortex^15^ integrates low-level sensory prediction errors with interoceptive and attentional expectations to regulate affective salience and emotion (Barrett and Simmons 2015; Seth 2013; Seth et al. 2012).

A closely related approach is the ‘embodied predictive interoceptive coding’ or EPIC model (Barrett and Simmons 2015; Chanes and Barrett 2016).^16^ EPIC begins from the viewpoint of hierarchical interoceptive processing proposed by IPC, which is then extended to a general RPP model of cortical function. On this account, the agranular ‘visceromotor’ cortex sits at the centre of the centrifugal hierarchy, modulating the precision or salience of perception and action across the hierarchy.^17^ In describing EPIC, Barrett and Simons appeal to a specific role of the insula, yet explicitly rule out a modular story, appealing instead to a connectionist (graph theoretical) view of the brain:

It may be tempting to view the interoceptive system, as outlined in the EPIC model, as a modular system. However, the brain has a small-world architecture... augmented by ‘rich-club’ hubs (that is, highly connected nodes), which ... serve as the brain’s ‘backbone’ for neural communication and synchrony. Several agranular visceromotor regions—including the anterior insula and cingulate cortices—are rich-club hubs, prompting the hypothesis that agranular visceromotor cortices send predictions to and receive prediction-error signals from cortices with greater laminar differentiation in an effort to create the kind of synchronized brain activity that is necessary for consciousness (Barrett and Simmons 2015, p. 425)

According to EPIC, bodily predictions act as a binding ‘pacemaker’ signal to create a core neuronal workspace synchronizing cortical representations to give rise to embodied, conscious awareness (Allen et al. 2016a; Dehaene et al. 2014). This notion of a predictive core or ‘embodied’ global neuronal workspace as the basis of the minimal self (Gallagher 2000) appears frequently in the embodied and interoceptive predictive coding literature, where it is typically leveraged to explain multisensory phenomenon such as the rubber-hand illusion, in which a conflict of exteroceptive and bodily signals results in a dynamic alteration of the body-schema (Apps and Tsakiris 2014; Limanowski and Blankenburg 2013; Park et al. 2014; Park and Tallon-Baudry 2014; Salomon et al. 2016; Suzuki et al. 2013).

In summary, interoceptive and embodied predictive coding models not only extend predictive coding to explain body-awareness; rather, they go beyond this weaker claim to argue that body-related predictions coordinate and contextualize global brain function. In other words;

The picture emerging here is one in which neural representations of the world that underlie perception and action are, in many cases, directed more by the homeostatic relevance of information than by the need for accuracy and completeness in representing the outside world. (Barrett and Simmons 2015, p. 7)

In support of this argument, recent evidence indicates that unexpected, unconscious surges of interoceptive arousal reverse the impact of sensory noise on perceptual awareness (Allen et al. 2016b). This inferential weighting of sensory representation by interoceptive precision is a clear departure from the strictly cognitivist stance described earlier. Instead of the brain being solely defined by veridical representation, perception and action are now fundamentally affective and embodied in nature, possessing a salience (epistemic) or inherent (pragmatic) value for the organism in homeostatic terms (see also Gallagher and Allen 2016). The brain is in the game of predicting the world, but only as a means to the end of embodied self-preservation. If this view is right, the Bayesian brain is only there to infer the right sorts of (epistemic or pragmatic) affordance necessary to predict the right sort of embodied engagement with the world.

Is this then the answer to our question; if cognition is defined according to the need to recover a hidden world through a generative model, can it also be embodied? Interoceptive predictive coding suggests that information is embodied in a contextualizing sense; internal signals encode the embodied state of the organism to imbue sensory perception and action with affective salience. One should exercise caution however; here, ‘embodiment’ is only in virtue of an internal (neuronal) model, which integrates interoceptive and exteroceptive ‘representations’. The ‘body’ in question is squarely inside the head—absent are the dense causal, dynamical couplings between internal and external phenomenon that characterize enactive views of embodied cognition (Chemero 2011; Gallagher 2000; Varela et al. 1991). Whether such representations are connectionist or modular in nature is of little concern; both views paint a homunculus into the picture.

Philosopher Alvin Goldman (Goldman 2012) provides a useful metric by which to characterize the spectrum of ‘embodiment’ found in predictive processing. Here Goldman proposed categorizing theories as ‘conservative’ (no embodiment at all, as in the case of strict modularity, nativism, etc), moderate or ‘lightly’ embodied (bodily information—encoded in B-formatted representations—influences cognition), or ‘radical’ (i.e., enactive and extended approaches arguing that the brain-body-environment is a dynamical system which constitutes cognition sans representation). Moderately embodied cognition thus invokes the ‘massive redeployment’ (c.f., Anderson 2007) of bodily representations (b-representations) to contextualize cognition:

Embodied cognition is a significant and pervasive sector of human cognition both because: (1) B-formats in their primary uses are an important part of cognition, and (2) B-formats are massively redeployed or reused for many other cognitive tasks, including tasks of social cognition. (Goldman 2012, p. 81)

This notion of b-representation clearly applies to ‘embodied predictive coding’ as described by its proponents. Information processing is done in the brain; perception, action, and cognition are ‘embodied’ insofar as they lend contextualizing information by body-format predictions (B-predictions) to cognition. Thus, although ‘embodied predictive coding’ may represent a productive compromise between embodied cognition and information processing, it is likely to leave proponents of more radical views unsatisfied.

## Escape from the body snatchers?

While the intermixing of b-representations and predictive coding motivates unique behavioural and neuroscientific hypotheses, we should also ask if something is lost in the appeal to B-predictions. Philosopher Shaun Gallagher provides a compelling critique,^18^ describing ‘moderate embodied cognition’ as an ‘invasion of the body-snatchers’:

... The body snatchers ...devise a version of embodied cognition that leaves the body out of it... Rather, the real action, all the essential action, occurs in the brain. Indeed, the body, in this version of embodied cognition, is the “body in the brain”. In effect, body snatchers have invaded theories of embodied cognition and have replaced bodies with “sanitised” body-formatted (or B-formatted) representations in the brain. (Gallagher 2015, pp. 97–98)

By appealing solely to the representational mixing of bodily and cognitive information, Gallagher suggests that proponents of ‘moderate’ embodied cognition actually argue for something that is not particularly embodied at all. Indeed, according to moderate (or weak) embodied cognition, such an agent could easily be simulated from within a vat, provided reasonable simulacra of bodily signals were provided. According to the moderate view, the body is just “a better designed container that delivers information to the brain in its own peculiar way”, rather than a constitutive element of information processing. What then should a radical theory of embodied prediction provide? Gallagher suggests that (bodily) anatomy, affect, sensory-motor contingencies, and environmental couplings—all of the things that weak EC reduces to neural representations—should be considered relevant to cognition.

Consider, then, the key features of embodied cognition as envisioned by its radical proponents; a causally constitutive role for sensorimotor contingencies, an enactive coupling of the organism to its body and environment, an ecological account of perceptual affordances, and a quality of affect and social meaning that pervades perception at the lowest level of information processing. Can predictive processing pass this high bar?

## Active inference & the free energy principle: bridging the divide

To resolve the tension between embodied cognition and predictive processing, we need to go beyond a mere description of the nervous system as an organ of prediction error minimization. Any account of cognition which appeals solely to predictive processing, will never fully escape the confines of the skull. For some, the moderate embodiment implied by interoceptive and embodied predictive coding is likely satisfactory, insofar as the framework provides an empirically testable model explaining how internal states breach modular encapsulation to lend affective warmth to perception. Yet, even allowing for this substantive progress, RPP (interoceptive or otherwise) are subject to a variety of critiques; which charge for example that they constitute circular/tautological reasoning (Bowers and Davis 2012), are unfalsifiable (Wiese 2014), and are merely convenient post-hoc or ‘just-so’ explanations (see Jones and Love 2011 for review, and various responses). Can enactivism save RPP from these pitfalls?

Indeed, the FEP tackles these issues head-on by providing a normative account of why—through active inference—the brain must necessarily engage in embodied predictive processing if it is to maintain its own enactive integrity. In doing so, the theory provides an empirical bridge between the computational and enactive views of the mind cashed out in terms of specific neuronal and embodied dynamics. To illustrate the link between these issues, consider that the commonly levied critiques (e.g., circularity, genesis of priors, and the definition of optimality) of RPP arise from a common problem; from what do the brain’s prior beliefs arise? This problem can be reformulated in a variety of ways. If our only imperative is to minimize prediction error, why do we not seek out the confines of a dark room? A simple solution is something like; because the brain has a prior which says “brains don’t like to be alone”. Here, we can see the circularity inherent to Bayesian decision theory; any behaviour can be described as optimal, because one can always write down a prior that prescribes any behaviour in a ‘just so’ fashion.^19^ For an acute example of this tautology, consider the case of reinforcement learning for values based-decision making, where a cost function guides ‘optimal’ behaviour, and cost is defined operationally by whatever an agent chooses.^20^

It is this ‘just so’ circularity that the FEP seeks to resolve by appeal to enactivism. Rather than the post-hoc definition of priors or cost functions, the FEP derives a normative, *a priori *first principle from a provable definition of living systems. To do so, the FEP highlights the necessary tendency of living organisms to resist the second law of thermodynamics; i.e., to maintain an internal structure or dynamics in the face of constant change. That is to say, by definition, living beings are those that maintain an upper bound on the entropy of their possible states. One can see this by considering a candle flame or snowflake; although both have some degree of persistent local dynamics, these do not resist the constant flux of the physical universe; they instead dissipate rapidly in the face of environmental fluctuations (a gust of air or the warmth of the sun). In contrast, to live is to visit some states more frequently than others—and visit their neighbourhoods time and time again (for example, our daily routine). However, before these imperatives can even be considered, the very existence of a system mandates the separation between the system and its external milieu (e.g., the environment in an evolutionary setting or heat bath in statistical physics). It is the separation or boundary that lies at the heart of the enactivist imperatives for predictive processing.

For example, a cell persists in virtue of its ability to create and maintain a boundary (cell-surface), through which it interacts with the environment, thereby maintaining the integrity of the boundary. It is this *autopoiesis*, or self-creation, which enables the system to limit the possible states it visits, and thus to survive (Varela et al. 1974). The FEP recasts this as a kind of self-fulfilling prophecy, in which an organism itself constitutes, in the generative sense, a belief that it will prevail within certain embodied and environmental conditions. In short, the very existence of a system depends upon conserving its boundary, known technically as a Markov blanket, so that it remains distinguishable from its environment—into which it would otherwise dissipate. The computational ‘function’ of the organism is here fundamentally and inescapably bound up into the kind of living being the organism is, and the kinds of neighbourhoods it must inhabit.

From this fundamental property of existence, it follows that any biological organism will possess the following characteristics:

- *Ergodicity* By placing an upper bound on entropy, an organism will necessarily occupy (the neighbourhood of) some states more often than others. This means that the average probability of being in a given state is equal to the probability of the system being in that state when observed at random. Note that this is simply a reformulation of the overall principle; to live (resp. be) is to revisit (resp. occupy) some characteristic states over time.
- *A Markov blanket* The boundary (e.g., between internal and external states of the system) can be described as a Markov blanket. The blanket separates external (hidden) from the internal states of an organism, where the blanket per se can be divided into sensory (caused by external) and active (caused by internal) states.
- *Active inference* The Markov blanket induces a circular causality because sensory states depend on hidden states that depend on active states, which depend upon internal states. In other words, the sensory and active states (that constitute the Markov blanket) mediate perception and action that are locked into a perpetual cycle to upper bound the entropy of both. Because the entropy of the Markov blanket is, by ergodicity, the time average of surprise or negative Bayesian log model evidence, sensory and active states will appear to maximise Bayesian model evidence. This means internal states can always be cast as representing external (hidden) causes—and thereby constitute a generative model of the causal forces that impinge upon them—while active states change the external states to make this job easier (e.g., avoid dark rooms).
- *Autopoiesis* Because active states change external (hidden) states, but are not changed by them, they will place an upper (free energy) bound on the entropy of biological states. This is because they are caused by internal states, and will therefore appear to maintain the structural and functional integrity of the internal states and their Markov blanket.

Simply put, an organism persists in virtue of having internal states which cause surprise-minimizing, evidence maximising actions; these in turn maintain the partitions described above, which is a necessary precondition for existence: c.f., the self-evidencing brain (Hohwy 2016). One can formulate this in another way; the organism’s internal states constitute probabilistic beliefs about what actions are the most likely to provide evidence for the organism’s existence (survival). My actions are not merely the output of an internal dynamic; the FEP argues that if I am to survive, they will actively bring about the conditions for my survival. The point is that the boundary itself is constituted by an ergodic dynamical interchange between ‘internal’ and ‘external’, rather than a cognitivist predominance of internal processing.

This notion is at the heart of autopoietic views of life and mind, insofar as it induces a deeply circular causality between internal and external states, to provide a normative principle by which to understand all action and perception. If an organism is endowed with the belief that it will maximize the evidence for its existence, then it will act in ways that are consistent with that belief. In other words, if survival is synonymous with minimizing surprise—i.e. maximizing evidence or self-evidencing (Hohwy 2016)—then it follows that the only possible prior belief an agent can entertain is that it will behave so as to minimize surprise. This is easy to see through reductio ad absurdum: if I believe I will be surprised, the only way I can be surprised is if I am not surprised. More exactly, the organism, body-brain-and-world itself constitutes the ‘belief’ or generative model that it will survive; in a very concrete sense, the kinds of limbs and morphological shape one has will constrain the probabilities of the kinds of actions one can engage in. This can be considered by analogy to the notion of an *Umwelt*, in which an organism’s world is itself a constituting and constraining feature of its embodiment (e.g., the isomorphism between the wavelength selectivity of our photoreceptors and ambient radiation from the sun).

This deep reciprocity between the embodied and environmental facts of the organism is embedded in the pattern of neural patterns which preconfigure the entity to best survive within its living world. Even seemingly ‘representationally hungry’ operations will be enmeshed within these looping, self-sustaining dynamics. For example, the organism will choose options that minimize its surprise, where free energy provides a tractable bound on surprise; hence the FEP. This bound is not absolute, computed solely in the head, but instead relative to the embodied nature of the organism as selected (via evolution) by the type of body and environmental niche inhabited by the organism. The implication is that my internal representations—the generative model of the world embodied in the web of neural connections—are causally coupled to my homeostatic needs and the environmental niche within which my brain has evolved.

Heuristically, this means that I will behave in ways consistent with my survival—which is itself consistent with or constrained by the type of body that I have, the econiche within which I have co-evolved, etc. If I am a cave bat, I will hang around in dark caves. If I am a human being, I will seek out other human beings and read articles on philosophy. The body itself is thus a prior boundary condition, or a conditioning factor, in the overall generative model defined by my Markov blanket. My body directly shapes my possibilities for (active) inference. The body-brain system has evolved to constitute a generative model, which specifies the types of behaviours, and environments in which I am likely to engage. Where one draws the boundaries is a matter of the question one wishes to ask; any living organism will be defined by a nearly infinite matryoshka embedding of blankets-within-blankets.^21^

The FEP thus provides a formal, information theoretic framework within which to explain the constitutive coupling of the brain to the body and the environment. The ‘cost function’ or imperative priors arise directly from the interoceptive, homeostatic needs of the body in exchange with the environment. My brain and body themselves constitutes a ‘belief’, in the generative sense about the kinds of states (e.g., homeostatic set points such as temperature, blood glucose) I must inhabit if I am to survive.^22^ The imperative to reduce free energy renders any action, which improves my survival inherently ‘desirable’—in the sense it brings me back to the attracting states prescribed by my generative model. Where, crucially, my self-evidencing generative model is learned or inherited from the environment; the capacities of my limbs for action preconfigure the nature of my active-inference.

Clearly, the active inference account satisfies the criteria for a radically embodied theory of mind. According to the free energy principle, an organism is best understood as a system of mutually interlocking systems; the body, mind and environment are inextricably bound up in the organism’s free energy minimization: in fact, all the heavy lifting done by active inference is in preserving a degree of (statistical) separation between the body, mind and environment (by maintaining the integrity of their respective Markov blankets). Perception is enactive and affective; through the reduction of surprise and uncertainty, perceptual and active states are selected to maximize the evidence for my existence. The body itself is a part and parcel of the computational machinery that leads to my survival. By elucidating these principles down in a formal, computational framework, the FEP provides an understanding of these issues that is amenable to experimentation and formal analysis. Although the FEP provides a normative, teleological essence to the synthesis of biology and information, the specifics of compliant (neuronal and behavioural) process theories must be discovered and verified empirically.

This is because, as a process theory, the specific couplings of action and body are left unspecified; which systems in the brain encode the uncertainty of some cognitive domain? What are the functional dissociations themselves; e.g., what solution has nature found to optimize the brain-body-econiche ensemble? In what specific ways do the affordances disclosed by these relations impact the cortical hierarchy, and vice-versa (Bruineberg and Rietveld 2014)? By providing much needed guideline to discovery (Chemero 2011), FEP renders a productive union of the embodied cognition and information theory, allowing the enactivist not only to describe the importance of the body, but to also build models of the brain-body-world relationship (See Friston et al. 2012a, b for one illustrative example).

FEP or active inference does not do this job for free; rather it provides a state theory, under which to develop specific process theories. One might here ask; if the FEP is unfalsifiable—in the sense that Hamilton’s principle of least action is not, in itself, falsifiable—is it uninformative? The FEP is uninformative in the sense that the principles of natural selection do not explain a particular species or phenotype; however, they inform the viability and sufficiency of any process theories. For example, expected utility theory and reinforcement learning are not sufficient theories of behaviour because they do not link utility or reward to free energy. Conversely, the theory of evolution by natural selection is free energy compliant (through its formulation as Bayesian model selection).

## Hiding beneath the Markov blanket

We note however, that not all are likely to agree with this interpretation. Some theorists take the Markov blanket to imply a strong partitioning, and this has led arguments directly contravening this type of claim; if my survival depends upon separating internal from external states, then I must infer through mental representation (i.e., Bayesian inference) the causal nature of those hidden, external states (Hohwy 2016).

There are at least two responses to this; first, the aim of the FEP, and the account sketched here, is not to deny the causal importance of the internal states. Indeed, an organism survives in virtue of its nervous system constituting (a partition of) a generative model, which can infer those actions most likely to maximize the evidence for its survival, as sampled indirectly through sensations. However, the delineation of the boundaries of a Markov blanket is essentially relative and variable; indeed any organism will be defined not by a singular blanket, but instead by a near-infinite regress of causally interacting Markov blankets within Markov blankets (and indeed of Markov blankets). In other words, the brain does not constitute a single blanket, but rather innumerable systems that are at once modular and dynamic, creating internal states in virtue of their interactions with one another. There will be a brain-body blanket, which describes the same circular causality. One can go still further; in an ecological sense, my ‘econiche’ or self-creating environment of tools and cultural settings—and my interaction within it—constitutes another blanket (See Bruineberg et al., this issue). Furthermore, blankets of blankets may change over many timescales; for example, with developmental cycles. This begs interesting questions about the timescales over which ergodicity applies—and whether these timescales are nested. Where one draws the causal demarcation is simply a matter of the question at hand. At all levels of description there will be a constant interaction between an emergent generative model and a coupled interaction thereof.

In short, the Markov blanket does not provide a cover from which to hide from external states in a radically sceptical fashion. On the other hand, the Markov blanket does not admit a radically realist position; in the sense a living system can never know what is ‘out there’—it can only infer, with a greater or lesser degree of accuracy, the causes of sensory impressions on the blanket. Perhaps it is best to construe Markov blankets as ‘gluing’ the brain to its body and the body to its econiche; however, by definition, this glue never comes with ‘phenomenologically transparent’ on the tin. This is not to say that the Markov blanket precludes direct perception in the sense of delivering an ‘optimal grip’ on the affordances offered by inferred external (hidden) states (Bruineberg and Rietveld 2014). In active inference, everything is in the service of predicting action, no matter how deep the hierarchical processing.^23^

Clearly, the explanatory scope of the FEP goes far beyond that of B-representations. Bodily information, and inference over those signals, is not only a contextual contributor. Rather, it is a part of the causal tapestry, which defines the agent and its viability as a ‘model’ of its environment. Another way to say this is: the causal machinery of the brain and its representations are enslaved within the brain-body-environment loop of autopoiesis—which is reminiscent of the circular causality that underwrites the slaving principle in synergetics (Haken 1977). This is an important point here; the FEP is not eliminating representations,^24^ nor is it hiding the body within the brain. Rather, the FEP directly explains the organism: the embodied brain becomes a model of its environment in virtue of exactly the kinds of autopoietic, dynamics espoused by proponents of radical embodiment.

## Conclusions and future questions

Predictive processing promises to revolutionize our understanding of how the brain—in conjunction with the body and environment—persists in the face of uncertainty. Here, we have reviewed a continuum of views; all of which can be gathered under predictive processing. Our intention was not to exhaustively cover all possible contributions; given the exponential growth of prediction-related theoretical and empirical work, such an effort would beyond the scope of the present issue. Instead, we have focused on how—rather than constituting a single, monolithic framework—predictive processing involves a wide range of theoretical commitments, with respect to the philosophy of mind. As we have shown, depending upon where one falls along this spectrum, competing modular, connectionist, or embodied/enactive theories can easily arise. Nevertheless, the FEP accommodates many of these disparate views within a single framework, whilst motivating novel empirical work.

Here we have sketched the enactive and embodied underpinnings of the FEP. Much work remains to be done on this issue. For example, although we here argue that the FEP resolves the tension between internalist and externalist approaches, we recognize that some may remain dissatisfied. We look forward to discourse concerning, for example, the notion of the brain-body-econiche as a kind of generative model, and whether one can sensibly speak of ‘representations’ under such a framework. If representation is distributed all around the brain and beyond, does it remain a representation in any meaningful sense? Similarly, many aspects of predictive processing suggest a role for ‘emergent’ or global processes (See Kim 1999 for critique), which constraints local processes in a kind of hermeneutic circle. It remains to be seen whether these are at tension with the account given here, and whether they lend themselves to a more cognitivist or representational view. Again, rather than delve deeply into these issues, our intention as to instead paint a general picture. For those who are interested, this special issue contains substantive views arguing for example, that the FEP does not require any ‘inference’ or ‘internal models’ (Bruineberg et al. this issue).

In summary, the FEP offers a formal path forward for enactivism. By providing a guideline to discovery, the normative principles embedded within the approach allow enactivists to go beyond arguing about the demarcations of the organism, to instead develop empirical theories of how brain, body, and world interact with one another. Ultimately it is this pragmatic ability to motivate testable hypothesis about ‘enactive computation’ that may most benefit cognitive science.

## Footnotes

1 The ability to accurately localize an object in external space depends not only the position of an object on the retina, but must also account for the orientation of the retina with respect to the orbit (i.e., gaze position).

2 As Helmholtz did, you can try this experiment for yourself; simply close one eye and (gently) press upon the eyelid to move the retina. The world will erroneously appear to move, suggesting that visual locations are determined using a prediction of eye position generated through efferent motor copy.

3 Though see Gallagher (2004) for a critique of the application of motor comparison to thought insertion.

4 For example, the sense of agency was hypothesized to depend upon the integration of motor feedback and intentional goals; experimentally, this corresponds to a hypothetical factorial interaction between these sub-components, and these can be experimentally manipulated independently of one another (Synofzik et al. 2008).

5 Certainly the strong emphasizes on internal forward models precludes them from any radical enactive or embodied theory. Hohwy goes further to argue that predictive processing deflates embodied and enactive cognition entirely (Hohwy 2016).

6 In some cases, precision is said to be encoded by the post-synaptic gain of neural circuits, as controlled by neuromodulators such as dopamine, serotonin, and acetylcholine (Bastos et al. 2012; Friston et al. 2012a, b; Moran et al. 2013).

7 Heuristically, one can think of this as a move to perception-as-inference (or synthesis) rather than by recollection.

8 As we shall see shortly, sometimes the essential dynamics include not only the central nervous system, but also peripheral and wider embodied organism (and beyond).

9 And potentially, the wider brain-body-environment organism, as we will argue shortly.

10 In some cases culture itself is consider a source of such ‘sub-optimal priors’. Note that any action or percept is only optimal insofar as it satisfies some prior belief, however ‘irrational’ that belief might be. This has been levied as an account of maladaptive (yet ‘optimal’) inference in psychosis (Friston et al. 2014a, b; Schwartenbeck et al. 2015).

11 Technically, these are empirical priors, if one regards the phenotype as the product of a model selection process that inherits prior constraints from a higher evolutionary level.

12 Presence denotes the feeling of veridicality that typically accompanies experience. For example, as I write this paper I have an implicit feeling that this is an actual experience in my waking life. The sense of presence is typically disrupted, for example, during hallucination or dreaming.

13 Interoception denotes the sensation and perception of homeostatic signals arising from the viscera (i.e., the heart, blood, gut, and lungs). Neurophysiologically, specialized peripheral and central nervous pathways communicate changes in blood pressure, glucose, heart rate, gut peristalsis, air hunger, and so on. These signals are projected principally to the posterior insula, which contains fine-grained visceral-maps (Craig 2003; Critchley and Harrison 2013).

14 Though crucially, with an emphasis on visceral, tactile, and nociceptive modalities (Craig 2003; Critchley and Harrison 2013).

15 The cingulate possesses similar pattern cellular and connectivity gradient, is also argued to play a similar, albeit more action-oriented role in conjunction with the insula.

16 See also recent work by Seth and colleagues, which expands IPC into a more general theory of sensorimotor contingencies and counter-factual prediction error (Seth 2014a, b).

17 Note that EPIC argues that viscero-motor cortices send ‘low-dimensionality predictions of predictions’, mathematically speaking these corresponded to expected precision (or hyper-priors).

18 See Gallagher (in preparation) for a more rigorous philosophical treatment of this issue.

19 Technically, this is known as the complete class theorem (Brown 1981).

20 Note the lack of falsifiability, which is inherent to any normative explanation in which I, as external observer, write down some condition for optimal behavior, rather than grounding that explanation in the necessary preconditions for the existence for that organism.

21 It is worth noting the strong resembles here to the epistemic view of enactivism as ‘mutually constrained, interlocking dynamics at all (bio/social/geo) levels envisioned by Varela (1991).

22 One can here pick up a debate about whether this notion of ‘belief’ resembles anything like the technical definition of ‘representations’ to which most enactivists object. Rather, they seem better suited to a dynamical system analysis. See Bruineberg et al., this issue for arguments on this issue.

23 This follows from the simple fact that action is the only way to underwrite an upper bound on the entropy of sensations. On the other hand, perceptual inference is the only way to inform action.

24 A possible argument here could be that it doesn’t have to since there are no such things, except in the eye of the external observer (scientist)—the brain itself doesn’t need them—or so the enactivist would say. So maybe one could say the FEP doesn’t eliminate the desire or need or feasibility or practicality on the part of the scientist to talk about representations, but instead renders a framework to understand the circular inter-relation between parts and whole, in a hermeneutic sense.
